# Dipeptidyl peptidase-4 inhibitor compared with sulfonylurea in combination with metformin: cardiovascular and renal outcomes in a propensity-matched cohort study

**DOI:** 10.1186/s12933-019-0835-z

**Published:** 2019-03-11

**Authors:** Kyoung Jin Kim, Jimi Choi, Juneyoung Lee, Jae Hyun Bae, Jee Hyun An, Hee Young Kim, Hye Jin Yoo, Ji A. Seo, Nan Hee Kim, Kyung Mook Choi, Sei Hyun Baik, Sin Gon Kim, Nam Hoon Kim

**Affiliations:** 1 Division of Endocrinology and Metabolism, Department of Internal Medicine, Korea University Anam Hospital, Korea University College of Medicine, 73, Inchon-ro, Seongbuk-gu, Seoul, 02841 Republic of Korea; 2 Department of Biostatistics, Korea University College of Medicine, 73, Inchon-ro, Seongbuk-gu, Seoul, 02841 Republic of Korea

**Keywords:** Cardiocerebrovascular disease, Dipeptidyl peptidase-4 inhibitors, End-stage renal disease, Heart failure, Sulfonylurea, Type 2 diabetes

## Abstract

**Background:**

To determine the impact of dipeptidyl peptidase-4 inhibitor (DPP4i) on the risk of major cardiocerebrovascular and renal outcomes compared with sulfonylurea (SU) combined with metformin in patients with type 2 diabetes from a population-based cohort.

**Methods:**

From a nationwide cohort in Korea (2008–2013), 23,674 patients with type 2 diabetes treated with DPP4i plus metformin or SU plus metformin were selected and matched by propensity score. Composite cardiocerebrovascular events including incident ischemic heart disease (IHD), ischemic stroke (IS), hospitalization for heart failure (HHF), and cardiocerebrovascular death, as well as renal events including incident end-stage renal disease or initiation of renal-replacement therapy were assessed by Cox proportional-hazards models.

**Results:**

During a median follow-up of 19.6 months (interquartile range 7.2–36.4), 762 composite cardiocerebrovascular events and 17 end-stage renal events occurred. There was no significant difference in the risk of IHD (hazard ratio [HR], 1.00; 95% CI 0.81–1.23), IS (HR, 0.95; 95% CI 0.74–1.23), or cardiocerebrovascular death (HR, 0.74; 95% CI 0.46–1.18) in the DPP4i group compared to that in the SU group. Likewise, DPP4i therapy was not associated with the risk of end-stage renal outcomes (HR, 1.23; 95% CI 0.41–3.62). However, the risk of HHF was significantly higher in the DPP4i group than in the SU group (HR, 1.47; 95% CI 1.07–2.04).

**Conclusions:**

This real-world database analysis showed that DPP4i therapy did not increase the overall risk of major cardiovascular and renal outcomes compared to SU therapy. However, the DPP4i-associated risk of HHF remained significant.

**Electronic supplementary material:**

The online version of this article (10.1186/s12933-019-0835-z) contains supplementary material, which is available to authorized users.

## Background

Dipeptidyl peptidase-4 inhibitors (DPP4i) are commonly used oral glucose-lowering agents that have intermediate efficacy with a low risk of hypoglycemia and neutral effects on body weight [1]. Previous cardiovascular outcome trials for DPP4i including the Examination of Cardiovascular Outcomes with Alogliptin versus Standard of Care (EXAMINE), Trial to Evaluate Cardiovascular Outcomes after Treatment with Sitagliptin (TECOS), and Saxagliptin Assessment of Vascular Outcomes Recorded in patients with diabetes mellitus-Thrombolysis in Myocardial Infarction-53 (SAVOR TIMI-53) have reported no significant increase in major adverse cardiovascular events (MACEs) compared to placebo [2–5]. Recently, the Cardiovascular and Renal Microvascular Outcome Study with Linagliptin (CAMELINA) study also has revealed a neutral effect of linagliptin on the risk of MACE as well as hospitalizations for heart failure (HHF) and composite renal outcome [6]. However, an exceptional warning signal of higher risk of hospitalizations for heart failure (HHF) of DPP4i was observed in the SAVOR TIMI-53 trial [4]. Since that, there have been a growing number of studies focusing on the association between DPP4i and risk of heart failure (HF). A meta-analysis suggested that DPP4i could increase the risk of HF [7]. Conversely, in a large observational study of incretin-based drugs, DPP4i was not associated with an increased risk of HHF in patients with type 2 diabetes, with or without a history of HF [8]. The other studies even indicated that DPP4i reduced the risk of HHF compared with other comparators such as sulfonylurea [9, 10].

In terms of the renal effects of DPP4i, there have been inconsistent results. Some studies have suggested that DPP4i therapy was beneficial, especially for inhibiting the progression of albuminuria in type 2 diabetes patients with or without chronic kidney disease (CKD) beyond their glucose-lowering effects [11–16]. The SAVOR-TIMI 53 trial also showed that saxagliptin treatment reduced the albumin creatinine ratio (ACR) in patients with various stages of CKD [17]. However, there was no clinically significant impact of sitagliptin on renal endpoints in the TECOS trial [18], and linagliptin did not reduce albuminuria compared to a placebo in the MARLINA‐T2D study [19].

Given that cardiovascular and renal outcomes are the most important endpoints in patients with type 2 diabetes, these inconsistencies demand further evidence for various settings. Meanwhile, DPP4i has been frequently prescribed in combination with metformin in clinical practice [20]. Therefore, the aim of the present study was to evaluate the effects of DPP4i therapy on cardiovascular and renal outcomes compared with SU as a comparator in combination with metformin within a population-based cohort.

## Methods

### Data source and patient selection

We employed a retrospective matched cohort design using the Korean National Health Insurance Service-Health Screening Cohort (NHIS-HEALS), which included 514,866 individuals aged between 40 and 79 years from the Republic of Korea. This represents 10% of a random selection within all of the health screening participants in the index year 2002 or 2003 and followed up through 2013. The NHIS requires all insured employees and self-employed persons more than 40 years as well as their dependents to participate in a general health screen every 2 years in order to improve the health status of Koreans through the prevention and early detection of disease. This database contains longitudinal information including the subjects’ demographics as well as medical and pharmaceutical records including disease code records according to the International Classification of Disease, Tenth Revision (ICD-10), medical procedures, hospitalization, information of prescribed drugs, and death records. The detailed cohort protocol has been described previously [21]. From the original database, we selected patients with type 2 diabetes (ICD-10 codes E11–14), who had received at least one oral glucose-lowering agent from December 1, 2008 (the date DPP4i was first released in Korea) to September 30, 2013 (the date when the results of SAVOR-TIMI 53 were released). Metformin, SU, thiazolidinedione (TZD), and DPP4i were included in this study as oral glucose-lowering agents. Among the included patients, we identified all of those who had been prescribed DPP4i or SU in combination with metformin. We chose SU as a comparator drug because SUs were one of the most frequently used second-line oral hypoglycemic agents added on metformin all over the world, especially under the Korean insurance. Furthermore, only patients who were initially treated with the study drugs (DPP4i and SU) were included in the study, and any patients who had received these agents alone or in other combinations before the index date were excluded. We also excluded patients who had previously used insulin formulations. Patients who died in the first month in the index year were also excluded. The index year was defined as the date when DPP4i or SU in combination with metformin were first prescribed.

The study subjects were divided into the following two groups: DPP4i group (DPP4i plus metformin) and SU group (SU plus metformin). The disposition of patients for the study is shown in Additional file 2: Figure S1. Two separate cohorts were created based on their underlying histories of cardiocerebrovascular disease (CVD) and HF, respectively. In the first cohort (CVD cohort), baseline CVD was defined by any former diagnosis of ischemic heart disease (IHD) (ICD codes I20–25) and cerebrovascular disease (ICD codes I60–64), or HF (ICD codes I50, I42–43). On the basis of the presence or absence of a recorded history of CVD, two subgroups were classified, matched and analyzed. In the second cohort (HF cohort), baseline HF was also defined as stated above. The analysis was performed in the same way as that in the first cohort. In the analysis for the renal outcomes, we selected only patients with available laboratory data for creatinine levels at baseline and had no history of end-stage of renal disease (ESRD) before the index year.

This study was approved by the institutional review board of the Korea University Anam Hospital.

### Outcome measures

The cardiocerebrovascular outcomes of interest were incident IHD (ICD-10 codes I20-I25 plus a procedure of coronary artery angiography), ischemic stroke (IS) (ICD-10 codes I63–66 with an examination of brain imaging study), hospitalization for HF (HHF; ICD-10 codes I50, I42 and I43), and death from CVD (ICD codes I00-I99). Composite CVD events included any of the components of CVD events. HHF event was defined as a primary or secondary diagnosis with ICD-10 codes mentioned above.

The end-stage renal outcomes were defined as having any of the following: diagnosis of ESRD (ICD-10 codes N18.0 and N18.5), hospital visits involving renal dialysis (Z49.1 and Z49.2), kidney transplantation status (Z94.0), procedures for hemodialysis or peritoneal dialysis (O7020, O7030-7034, O7071, and O7072), or surgical procedures for kidney transplantation (R3280).

Each patient was followed up from the index date up to the earliest occurrence of any study outcomes, discontinuation of pre-specified regimens (the study drug was stopped, or the alternative study drug was added), death, or the end of the study period (September 30, 2013).

### Confounder variables

Confounder variables measured for this study included the patient age at index date, gender, duration of diabetes, fasting blood sugar (FBS), body mass index (BMI), systolic blood pressure (SBP), and previous prescribed use of TZD. Prescribed drugs including antihypertensive medication, statins, antiplatelet agents, and anticoagulation agents were regarded as those used more than 30 days before the index year. Due to the fact that adjustment of too many similar variables can cause conflict, we chose medications for hypertension and dyslipidemia instead of ICD codes. Diabetes duration (in years) was assessed from the date of the first medical treatment for a diagnosis of diabetes until the date of an event. The patients’ histories of smoking, alcohol consumption and physical activity were also adjusted. For statistical analyses, these data were classified further into three groups as follows: smoking (never, former, or current smokers), alcohol consumption (none, twice per week, or ≥ three times per week), and physical activity (none, ≤ twice per week, or ≥ three times per week).

### Statistical analyses

Continuous variables were presented as mean ± standard deviation (SD), and the categorical variables were described as numbers with percentages. Cumulative incidence rates and 95% confidence intervals (CI) for the study outcome were calculated using the Kaplan–Meier method. The propensity score matching (PSM) and inverse probability of treatment weighting (IPTW) were used to reduce the effects of confounders between the DPP4i and SU groups. The propensity score defining each individual’s probability of receiving the DPP4i plus metformin treatment was developed by a multiple logistic regression model that included all of the variables in both cohorts mentioned in Additional file 1: Tables S1 and S2 in both cohorts: index year, age, sex, duration of diabetes, fasting blood sugar, ever-prescription for TZD, BMI, SBP, prescribed drugs, and histories of smoking, alcohol consumption, and physical activity. For the PSM, we performed a 2:1 matching (two cases per one control patient within strata based on the presence or absence of baseline CVD or HF. Baseline characteristics between the two groups were compared using generalized mixed models with appropriate link functions in each matched cohort. A stratified Cox’s proportional hazards regression analysis for matched data was performed to evaluate the relative hazard of events in the DPP4i group compared to that in the SU group after adjusting covariates used in the multiple logistic regressing model. We also used IPTW, which is a powerful tool for observational data [22]. The weights based on each individual’s propensity score were calculated by the inverse of the score in the DPP4i group and the inverse of 1 minus the score in the SU group [23]. We then estimated the hazard ratio (HR) and 95% CI from the weighted Cox’s proportional hazards regression analysis using IPTW. A *P* value < 0.05 was considered statistically significant. All statistical analyses were performed using SAS software version 9.4 (SAS Institute Inc., Cary, NC, USA).

## Results

### Study population

The cohort included a total of 23,635 patients; 16,803 patients were treated with a DPP4i plus metformin, and 6832 were treated with a SU plus metformin (Additional file 2: Figure S1). The mean age of study subjects was 62 years, and 61% were women. 4.2% had been treated with TZD before the index date. The frequencies of statins and anti-thrombotics prescribed were 49% and 41%, respectively. Additional file 1: Table S1 describes the baseline characteristics of the DPP4i group (*n *=9368) and SU group (*n *=4684) according to the baseline CVD (1st cohort), which were well balanced after PSM. An additional table showing the baseline characteristics of the 2nd cohort according to the baseline HF also demonstrated well-matched profiles between the groups (Additional file 1: Table S2).

During a median follow-up of 19.6 months (interquartile range 7.2–36.4), 762 composite CVD events and 17 cases of ESRD occurred in the 1st cohort. In the 2nd cohort, there were 201 HHF events and 28 cases of ESRD during a median follow-up of 19.3 months (interquartile range 7.1–36.4).

### Cardiocerebrovascular outcomes

The composite and individual CVD events were analyzed by a multiple logistic regression model (Table 1). Because the number of patients followed after 3 years were largely reduced due to changes of initial treatment regimens, we analyzed the risks separately for the 3rd and 5th years.

**Table 1:** Relative risks of CVD and ESRD in SU group vs. DPP4i group (1st cohort)

Study outcomes	Total			History of baseline CVD			No history of baseline CVD		
	SU + MET (*n* = 4684)	DPP4i + MET (*n* = 9368)	*P*-value	SU + MET (*n* = 2025)	DPP4i + MET (*n* = 4050)	*P*-value	SU + MET (*n* = 2659)	DPP4i + MET (*n* = 5318)	*P*-value
Composite CVD events^a^									
*N*. of events	300	462		247	361		53	101	
Cumulative incidence at 3 years (%)^b^	8.06 (7.14–9.10)	8.01 (7.23–8.87)		15.23 (13.39–17.28)	14.08 (12.60–15.73)		2.36 (1.75–3.19)	3.36 (2.68–4.21)	
HR (95% CI) at 3 years^c^	1.00	1.02 (0.88–1.19)	0.7702	1.00	0.99 (0.83–1.174)	0.8657	1.00	1.32 (0.92–1.90)	0.1370
Cumulative incidence at 5 years (%)^b^	11.33 (9.90–12.95)	13.73 (10.96–17.13)		20.07 (17.59–22.86)	24.15 (18.72–30.83)		4.20 (2.84–6.19)	5.07 (3.84–6.67)	
HR (95% CI) at 5 years^c^	1.00	1.04 (0.90–1.20)	0.6209	1.00	1.01 (0.86–1.18)	0.9309	1.00	1.29 (0.92–1.81)	0.1357
IHD									
*N*. of events	144	215		120	163		24	52	
Cumulative incidence at 3 years (%)^b^	4.05 (3.38–4.83)	3.79 (3.25–4.41)		7.93 (6.54–9.59)	6.66 (5.61–7.90)		1.07 (0.68–1.66)	1.66 (1.20–2.28)	
HR (95% CI) at 3 years^c^	1.00	0.99 (0.80–1.24)	0.9490	1.00	0.93 (0.73–1.19)	0.5846	1.00	1.39 (0.82–2.35)	0.2193
Cumulative incidence at 5 years (%)^b^	5.53 (4.56–6.70)	5.39 (4.49–6.45)		10.37 (8.54–12.57)	8.72 (7.23–10.51)		1.76 (0.99–3.12)	2.87 (1.91–4.32)	
HR (95% CI) at 5 years^c^	1.00	1.00 (0.81–1.23)	0.9713	1.00	0.93 (0.73–1.17)	0.5183	1.00	1.45 (0.89–2.35)	0.1350
IS									
*N*. of events	99	137		77	111		22	26	
Cumulative incidence at 3 years (%)^b^	2.62 (2.11–3.25)	2.38 (1.96–2.90)		4.47 (3.70–5.96)	4.39 (3.55–5.44)		0.99 (0.61–1.61)	0.85 (0.53–1.34)	
HR (95% CI) at 3 years^c^	1.00	0.89 (0.68–1.17)	0.4095	1.00	0.93 (0.68–1.25)	0.6102	1.00	0.81 (0.44–1.51)	0.5125
Cumulative incidence at 5 years (%)^b^	3.80 (2.97–4.87)	4.77 (3.11–7.30)		6.16 (4.82–7.85)	9.02 (5.48–14.64)		1.96 (1.04–3.66)	1.38 (0.81–2.32)	
HR (95% CI) at 5 years^c^	1.00	0.95 (0.74–1.23)	0.6956	1.00	0.98 (0.74–1.31)	0.9070	1.00	0.80 (0.45–1.42)	0.4407
HHF									
*N*. of events	65	128		56	103		9	25	
Cumulative incidence at 3 years (%)^b^	1.63 (1.23–2.17)	2.26 (1.85–2.74)		3.34 (2.48–4.48)	3.98 (3.20–4.93)		0.28 (0.12–0.68)	0.95 (0.62–1.47)	
HR (95% CI) at 3 years^c^	1.00	1.47 (1.07–2.04)	0.0186	1.00	1.30 (0.92–1.84)	0.1425	1.00	3.32 (1.28–8.62)	0.0139
Cumulative incidence at 5 years (%)^b^	2.90 (2.16–3.89)	4.43 (2.55–7.66)		5.29 (3.87–7.21)	8.63 (4.74–15.43)		0.94 (0.44–1.99)	0.95 (0.62–1.47)	
HR (95% CI) at 5 years^c^	1.00	1.34 (1.00–1.81)	0.0495	1.00	1.26 (0.91–1.74)	0.1717	1.00	1.91 (0.93–3.93)	0.0777
CVD death									
*N*. of events	35	36		31	29		4	7	
Cumulative incidence at 3 years (%)^b^	0.90 (0.62–1.31)	0.60 (0.41–0.88)		1.86 (1.26–2.75)	1.13 (0.74–1.73)		0.14 (0.04–0.44)	0.20 (0.09–0.47)	
HR (95% CI) at 3 years^c^	1.00	0.68 (0.41–1.14)	0.1443	1.00	0.62 (0.36–1.09)	0.0983	1.00	1.25 (0.30–5.10)	0.7595
Cumulative incidence at 5 years (%)^b^	1.45 (0.98–2.13)	1.24 (0.76–2.04)		2.85 (1.91–4.24)	2.41 (1.39–4.14)		0.29 (0.09–0.94)	0.30 (0.13–0.70)	
HR (95% CI) at 5 years^c^	1.00	0.74 (0.46–1.18)	0.2023	1.00	0.67 (0.40–1.12)	0.1281	1.00	1.17 (0.34–4.02)	0.8079

Study outcomes	Total			History of baseline CVD			No history of baseline CVD		
	SU + MET (***n*** = 4060)	DPP4i + MET (***n*** = 7618)	P-value	SU + MET (n = 1672)	DPP4i + MET (n = 3143)	P-value	SU + MET (*n* = 2388)	DPP4i + MET (*n* = 4475)	P-value
ESRD events^d^									
*N*. of events	7	10		5	5		2	5	
Cumulative incidence at 3 years (%)^b^	0.21 (0.08–0.52)	0.20 (0.10–0.39)		0.28 (0.08–0.92)	0.25 (0.10–0.61)		0.16 (0.04–0.64)	0.16 (0.06–0.45)	
HR (95% CI) at 3 years^c^	1.00	1.23 (0.42–3.62)	0.7082	1.00	1.28 (0.32–5.17)	0.7253	1.00	1.41 (0.27–7.41)	0.6860
Cumulative incidence at 5 years (%)^b^	0.48 (0.19–1.23)	0.26 (0.13–0.52)		0.87 (0.29–2.59)	0.25 (0.10–0.61)		0.16 (0.04–0.64)	0.27 (0.10–0.73)	
HR (95% CI) at 5 years^c^	1.00	1.02 (0.40–2.63)	0.9368	1.00	0.91 (0.28–2.93)	0.8686	1.00	1.80 (0.36–9.13)	0.4765

All of cardiovascular and renal outcomes were assessed using a Cox proportional hazards models comparing dipeptidyl-peptidase 4 inhibitor with sulfonylurea in combination with metformin after propensity score matching (PMS). PSM was performed by an optimal 2:1 (case: control) matching within a radius of 0.01

CVD, cardiocerebrovascular disease; DPP-4 inhibitor, dipeptidyl peptidase-4 inhibitor; *N*, number; HR, hazard ratio; CI confidence interval; IHD, ischemic heart disease, IS, ischemic stroke, HHF, hospitalization for heart failure; and ESRD, end-stage renal disease

^a^Any occurrence of IHD, IS, HF, or CVD death

^b^Cumulative incidence was calculated based on Kaplan–Meier estimation

^c^*P*-value by cox proportional regression model for clustered data

^d^Adjusted for creatinine

**Fig. 1:**
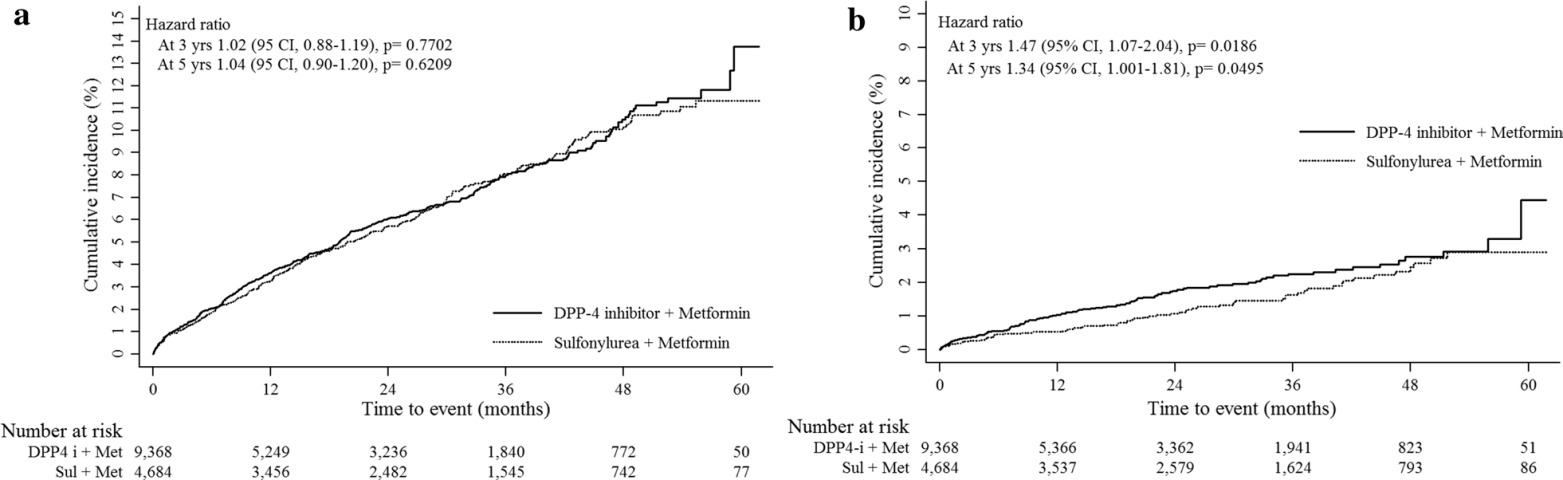
Comparison of cumulative incidence for cardiocerebrovascular disease (CVD) outcomes according to the baseline CVD. **a** Incidence of total composite cardiovascular events. **b** Incidence of hospitalization for heart failure

In the 1st cohort, DPP4i plus metformin therapy was not associated with an increased risk of composite CVD events (3rd year: HR, 1.02; 95% CI 0.88–1.19; 5th year: HR, 1.04; 95% CI 0.90–1.20) compared to SU plus metformin. The HRs of IHD, IS, and CVD deaths in the DPP4i group were 0.99 (95% CI 0.80–1.24), 0.89 (95% CI 0.68–1.17) and 0.68 (95% CI 0.41–1.14), respectively, in the 3rd year; and 1.00 (95% CI 0.81–1.23), 0.95 (95% CI 0.74–1.23) and 0.74 (95% CI 0.46–1.18), respectively, in the 5th year. However, the risk of HHF was significantly higher in the DPP4i group (3rd year: HR, 1.47; 95% CI 1.07–2.04; 5th year: HR, 1.34; 95% CI 1.01–1.81) than in the SU group (Table 1, Fig. 1). In the subgroup with no baseline CVD, the increased risk of HHF remained significant in the DPP4i group in the 3rd year (HR, 3.32; 95% CI 1.28–3.32). However, there were no significant differences in HHF between the groups in the subgroup with baseline CVD. From the 2nd cohort, DPP4i-related risk of HHF was also significantly higher in the 3rd year (HR, 1.39; 95% CI 1.02–1.90), but not in the 5th year (HR, 1.26; 95% CI 0.95–1.67) (Table 2; Additional file 2: Figure S2A). Further subgroup analyses for the risk of HHF was performed according to baseline CKD (Additional file 1: Table S3), history of TZD use (Additional file 1: Table S4 and Table S5) and stratified by individual DPP4 inhibitors (Additional file 1: Table S6), which resulted in similar findings of DPP4i-associated increased risk of HHF. However, those results are limited by small numbers of HHF events in each subgroups.

**Table 2:** Relative risks of HHF and ESRD in SU group vs. DPP4i group (2nd cohort)

Study outcomes	Total			History of baseline HF			No history of baseline HF		
	SU + MET (*n* = 4674)	DPP4i + MET (*n* = 9348)	*P*-value	SU + MET (*n* = 412)	DPP4i + MET (*n* = 824)	*P*-value	SU + MET (*n* = 4262)	DPP4i + MET (*n* = 8524)	*P*-value
HHF									
*N*. of events	70	131		44	73		26	58	
Cumulative incidence at 3 years (%)^a^	1.70 (1.30–2.23)	2.20 (1.82–2.67)		11.08 (8.00–15.26)	13.40 (10.53–16.97)		0.73 (0.47–1.15)	1.09 (0.81–1.47)	
HR (95% CI) at 3 years^b^	1.00	1.39 (1.02–1.90)	0.0369	1.00	1.29 (0.86–1.95)	0.2250	1.00	1.61 (0.97–2.67)	0.0634
Cumulative incidence at 5 years (%)^a^	2.97 (2.25–3.91)	3.30 (2.46–4.44)		19.96 (14.24–27.58)	19.44 (12.38–29.79)		1.21 (0.77–1.91)	1.68 (1.15–2.45)	
HR (95% CI) at 5 years^b^	1.00	1.26 (0.95–1.67)	0.1132	1.00	1.12 (0.77–1.64)	0.5574	1.00	1.51 (0.96–2.39)	0.0765

Study outcomes	Total			History of baseline HF			No history of baseline HF		
	SU + MET (*n* = 4066)	DPP4i + MET (*n* = 7635)	*P*-value	SU + MET (*n* = 333)	DPP4i + MET (*n* = 613	P	SU + MET (*n* = 3733)	DPP4i + MET (*n* = 7022)	P-value
ESRD events^c^									
*N*. of events	11	17		2	4		9	13	
Cumulative incidence at 3 years (%)^a^	0.34 (0.16–0.73)	0.42 (0.25–0.70)		0.00	1.23 (0.43–3.51)		0.37 (0.17–0.81)	0.35 (0.20–0.63)	
HR (95% CI) at 3 years^b^	1.00	1.55 (0.65–3.71)	0.3255		–		1.00	1.17 (0.47–2.92)	0.7298
Cumulative incidence at 5 years (%)^a^	0.77 (0.38–1.54)	0.48 (0.29–0.80)		1.81 (0.45–7.11)	1.23 (043–3.51)		0.67 (0.31–1.48)	0.42 (0.23–0.75)	
HR (95% CI) at 5 years^b^	1.00	1.10 (0.54–2.28)	0.7905	1.00	1.81 (0.45–7.11)	0.3977	1.00	1.04 (0.46–2.35)	0.9313

All of cardiovascular and renal outcomes were assessed using a Cox proportional hazards models comparing dipeptidyl-peptidase 4 inhibitor with sulfonylurea in combination with metformin after propensity score matching (PMS). PSM was performed by an optimal 2:1 (case: control) matching within a radius of 0.01

HF, heart failure; DPP-4 inhibitor, dipeptidyl peptidase-4 inhibitor; HHF, hospitalization for heart failure; *N*, number; HR, hazard ratio; CI confidence interval; and ESRD, end-stage renal disease

^a^Cumulative incidence was calculated based on Kaplan–Meier estimation

^b^*P*-value by cox proportional regression model for clustered data

^c^Adjusted for creatinine

In the model of IPTW (Additional file 1: Table S7), the HRs of HHF were 1.59 (95% CI 1.16–2.17) in the 3rd year and 1.36 (95% CI 1.00–1.87) in the 5th year. These trends were maintained in subgroups both with and without baseline CVD.

### Renal outcomes

**Fig. 2:**
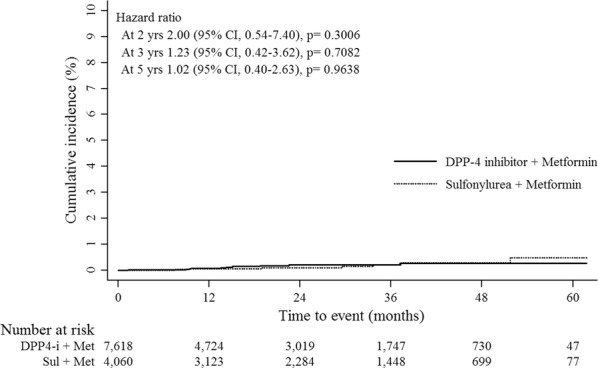
Comparison of cumulative incidence for renal outcomes according to the baseline cardiocerebrovascular disease (CVD)

For renal outcomes, we included patients with data on creatinine and without a previous diagnosis of ESRD. The median values of creatinine were 1.05 in DPP-4i group, and 1.01 in SU group (p = 0.01). The risk of ESRD was not higher in the DPP4i group than in the SU group. In the 1st cohort, the HRs were 1.23 (95% CI 0.42–3.62) in the 3rd year, and 1.02 (95% CI 0.40–2.63) in the 5th year (Table 1, Fig. 2). The results in the 2nd cohort were similar to those of the 1st cohort (3rd year: HR, 1.55; 95% CI 0.65–3.71; 5th year: HR, 1.10; 95% CI 0.54–2.28) (Table 2; Additional file 2: Figure S2B).

## Discussion

In this population-based cohort study analyzed using PSM methods, DPP4i in combination with metformin did not increase the risk of fatal or non-fatal atherosclerotic CVD and end-stage renal events compared with SU in combination with metformin in patients with type 2 diabetes. However, DPP4i therapy was associated with a higher risk of HHF regardless of underlying CVD or HF.

Substantial evidence has suggested beneficial cardiovascular effects of DPP4i including preservation of left ventricular function, decreasing myocyte apoptosis, diminishing oxidative stress, and improvement of endothelial function in experimental models as well as lowering blood pressure and improving lipid profiles in clinical trials [24–28]. Some observational studies revealed that DPP4i might lower HHF risk compared with SU [9, 10, 29–31] and others have shown a neutral effect on HHF with DPP4i [32–34]. A nationwide cohort study showed that DPP4i therapy even contributed to the improvement in survival after first acute myocardial infarction [35]. They provided several benefits of the DPP4i related to cardiovascular outcomes; DPP4i may reduce reperfusion injury and oxidative stress in addition to inhibiting cardiac dysfunction and adverse remodeling in the post-myocardial infarction settings.

On the other hands, there have been growing evidence for the increased risk for HF related to DPP4i. Aside from the SAVOR-TIMI 53 trial, a post hoc analysis of the EXAMINE trial also revealed the potential risk of HHF with alogliptin in patients without baseline HF [36]. The mechanism of increased HHF risk with DPP4i therapy has remained unresolved and not fully understood [37, 38]. There has been no direct evidence in humans demonstrating that DPP-4 inhibition directly affects heart function or retains fluid [37]. However, several studies have supported the increased risk of HHF by DPP4i. Un-cleaved brain natriuretic peptides, which are known as substrates of the enzyme DPP-4, might be associated with decompensated HF [38, 39]. Previous studies also suggested that upregulation of stromal cell-derived factor-1α may play a role in increased vascular permeability states such as retinopathy and HF [40, 41]. A study about patients with reduced left ventricular function revealed that treatment with DPP4i increased left ventricular end diastolic volumes [42]. These findings were consistent with our results.

It also should be noted that DPP4i-associated risk of HHF was prominent in patient without history of baseline CVD compared to in patients with history of baseline CVD (HR, 3.32 vs. 1.47). Similar findings were observed in the previous cardiovascular outcome trials; in the post hoc analysis of the SAVOR-TIMI trial [43], the risk of HHF with saxagliptin was significant in patients with no prior diagnosis of HF (HR, 1.32; p = 0.02), but not in those with prior diagnosis of HF (HR, 1.23; p = 0.13). Also in the EXAMINE trial post hoc analyses [36], increased risk of HHF was observed only in patients without history of HF at baseline, thereby indirectly supported our present findings. Nevertheless, our data do not offer clear explanation, and more direct evidence is needed for clarification of these results.

Considering the differences in design, population, and comparisons among the available studies, our study was strengthened by the reflection of real clinical practice regimens with well-matched comparators. We also showed that the DPP4i-related risk of HHF was maintained in different subgroups and across different analytical methods. Moreover, we analyzed the available data up to the year 2013 when the SAVOR-TIMI 53 study was published, which are the merits of our study compared to other studies. Other Asian studies had quite a similar design to ours, but their durations exceeded that of the influential SAVOR-TIMI 53, which first raised concerns of increased hospitalizations for patients with HF [10, 38, 39]. Therefore, our study did not have any possible bias regarding changes in treatment behavior following safety concerns. Another important point to address is that for the majority of participants in this study, the DPP4i used was sitagliptin (60.5%), followed by vildagliptin (25.78%). As shown in an additional table (Additional file 1: Table S8) saxagliptin was only administered to 1.97% of patients. In addition, in the analyses for the individual DPP4is, generally increased risk of HHF was observed in the DPP4i groups, especially sitagliptin (Additional file 1: Table S6). This result appeared to be directly contrary to TECOS [3]. Fadini et al. also reported that there was no intraclass difference for DPP4is related to HHF [44]. Therefore, it still remains unclear whether HHF risk is a class effect of DPP4is or not.

In the present study, the risk of ESRD was comparable between the DPP4i and SU groups. The mechanism involved in the albuminuria-lowering effect by DPP4i may be explained by both GLP-1-dependent and GLP-1-independent pathways as well as by improvements in hyperglycemia. For example, increasing GLP-1 concentration by DPP-4 inhibition had a protective role against oxidative stress through the inhibition of NAD(P)H oxidase and cAMP-dependent protein kinase pathway activation [45]. DPP4i also may reduce urinary albumin excretion by inhibition of tumor necrosis factor-α [46] and by reduction of osteopontin levels [12, 46]. However, there are limited data available indicating that DPP4i had beneficial effects on more advanced stages of kidney disease. None of the previous cardiovascular outcome trials, TECOS, EXAMINE, and SAVOR-TIMI demonstrated a reduction of ESRD risk in terms of initiation of dialysis or renal transplant, which were consistent with our results [2, 11, 17, 18]. The CARMELINA study showed that linagliptin treatment was beneficial for albuminuria progression compared to placebo, however, this study also did not prove the beneficial effect on the ESRD. We should wait for the direct evidence from future clinical trials for a more definitive conclusion.

There were several limitations to this study. First, we were not able to assess all of the confounding factors including HbA1c levels due to a lack of relevant data. HbA1c must be one of the most important factors in the management of diabetes including decision to combination therapy or insulin therapy. So, the limitation should be overcome by further studies with relevant data. Instead, we attempted to adjust for age, mean fasting glucose levels, and the duration of diabetes. Second, the study period was relatively short to fully assess the long-term outcomes. Third, the number of patients followed up slowly decreased in the latter part of the study, leading to narrowing a gap in the outcomes between groups. This was because the opposite drug was added. However, quite a large number of patients were still involved in the 3rd year relative to the index data; thus, we focused our analysis on the 3rd year rather than the 5th year.

## Conclusion

Our findings indicated that DPP4i therapy was not associated with a risk of major cardiocerebrovascular and renal events compared to SU in patients with type 2 diabetes; however, DPP4i therapy was associated with increased risk of HHF. To better clarify this issue, ongoing clinical trials directly comparing DPP4i with SU may provide more conclusive information.

## Additional files

**Additional file 1.** Additional tables.

**Additional file 2: Figure S1.** Disposition of study subjects. **Figure S2.** Comparison of cumulative incidence for CVD outcomes according to the baseline HF. (A) Incidence of hospitalization for heart failure. (B) Incidence for end-stage renal disease events.

## Abbreviations

CKD: chronic kidney disease
CVD: cardiocerebrovascular disease
DPP4i: dipeptidyl peptidase-4 inhibitor
ESRD: end-stage renal disease
HHF: hospitalization for heart failure
HF: heart failure
ICD-10: International Classification of Disease, Tenth Revision
IHD: ischemic heart disease
IPTW: inverse probability of treatment weighting
IS: ischemic stroke
MI: myocardial infarction
PSM: propensity score matching
SU: sulfonylurea
TZD: thiazolidinedione
