# Supplementary material for: Dipeptidyl peptidase-4 inhibitor compared with sulfonylurea in combination with metformin: cardiovascular and renal outcomes in a propensity-matched cohort study
Source: Cardiovasc Diabetol. 2019 Mar 11;18:28. doi: 10.1186/s12933-019-0835-z (PMC6410523; doi:10.1186/s12933-019-0835-z)
Supplement: Supplementary file 1 — Additional file 1. Additional tables. [file 12933_2019_835_MOESM1_ESM.docx]

**Online-Only Additional Tables**

**Table S1.** Baseline characteristics of cases and matched controls according to the presence or absence of a history of cardiovascular disease (CVD) by a propensity score matching method (1^st^ cohort)

| **Characteristics** | **Total** | | | **History of baseline CVD** | | | **No history of baseline CVD** | | |
| --- | --- | --- | --- | --- | --- | --- | --- | --- | --- |
|  | DPP-4i  + MET  (*n* = 9,368) | SU  + MET^a^  (*n* = 4,684) | *P*-value^b^ | DPP-4i  + MET  (*n* = 4,050) | SU  + MET^a^  (*n* = 2,025) | *P*-value^b^ | DPP-4i  + MET  (*n* = 5,318) | SU  + MET^a^  (*n* = 2,659) | *P*-value^b^ |
| Index year, *n* (%) |  |  | 0.822 |  |  | 0.9097 |  |  | 0.8612 |
| 2008 | 107 (1.1) | 60 (1.3) |  | 47(1.2) | 27 (1.3) |  | 60 (1.1) | 33 (1.2) |  |
| 2009 | 2,166 (23.1) | 1,058 (22.6) |  | 1,048 (25.9) | 520 (25.7) |  | 1,118 (21.0) | 538 (20.2) |  |
| 2010 | 2,107 (22.5) | 1,056 (22.5) |  | 964 (23.8) | 476 (23.5) |  | 1,143 (21.5) | 580 (21.8) |  |
| 2011 | 1,908 (20.4) | 972 (20.8) |  | 781 (19.3) | 390 (19.3) |  | 1,127 (21.2) | 582 (21.9) |  |
| 2012 | 1,789 (19.1) | 900 (19.2) |  | 722 (17.8) | 372 (18.4) |  | 1,067 (20.1) | 528 (19.9) |  |
| 2013 | 1,291 (13.8) | 638 (13.6) |  | 488 (12.0) | 240 (11.9) |  | 803 (15.1) | 398 (15.0) |  |
| Age, mean (SD) | 62.3 (9.0) | 62.5 (9.2) | 0.1081 | 64.9 (9.1) | 65.1 (9.3) | 0.3309 | 60.4 (8.4) | 60.5 (8.6) | 0.1709 |
| Age group, *n* (%) |  |  | 0.365 |  |  | 0.4817 |  |  | 0.5815 |
| 46–49 | 391 (4.2) | 223 (4.8) |  | 112 (2.8) | 59 (2.9) |  | 279 (5.2) | 164 (6.2) |  |
| 50–59 | 3,654 (39.0) | 1,787 (38.2) |  | 1,144 (28.2) | 568 (28.0) |  | 2,510 (47.2) | 1,219 (45.8) |  |
| 60–69 | 3,088 (33.0) | 1,488 (31.8) |  | 1,414 (34.9) | 689 (34.0) |  | 1,674 (31.5) | 799 (30.0) |  |
| 70–79 | 1,913 (20.4) | 995 (21.2) |  | 1,170 (28.9) | 575 (28.4) |  | 743 (14.0) | 420 (15.8) |  |
| ≥80 | 322 (3.4) | 191 (4.1) |  | 210 (5.2) | 134 (6.6) |  | 112 (2.1) | 57 (2.1) |  |
| Sex, *n* (%) |  |  | 0.9026 |  |  | 0.8252 |  |  | 0.9587 |
| Male | 5,728 (61.1) | 2,859 (61.0) |  | 2,247 (55.5) | 1,117 (55.2) |  | 3,481 (65.5) | 1,742 (65.5) |  |
| Female | 3,640 (38.9) | 1,825 (39.0) |  | 1,803 (44.5) | 908 (44.8) |  | 1,837 (34.5) | 917 (34.5) |  |
| Duration of diabetes, mean (SD), years | 6.4 (3.2) | 6.3 (3.3) | 0.0568 | 7.0 (3.0) | 6.9 (3.2) | 0.2908 | 6.0 (3.2) | 5.9 (3.4) | 0.1020 |
| FBS,  mean(SD),mg/dL | 126.0 (29.1) | 125.6 (31.1) | 0.3469 | 120.8 (25.4) | 120.3 (28.2) | 0.4726 | 130.0 (31.1) | 129.6 (32.6) | 0.5024 |
| Use of TZD, *n* (%) | 392 (4.2) | 197 (4.2) | 0.9625 | 179 (4.4) | 80 (4.0) | 0.3933 | 213 (4.0) | 117 (4.4) | 0.4109 |
| BMI,  mean (SD), kg/m^2^ | 25.3 (3.0) | 25.3 (3.0) | 0.6353 | 25.5 (3.1) | 25.5 (3.1) | 0.8737 | 25.1 (2.9) | 25.1 (2.9) | 0.6303 |
| SBP,  mean (SD), mmHg | 130.5 (12.4) | 130.7 (12.0) | 0.6137 | 132.0 (12.6) | 132.2 (12.1) | 0.6900 | 129.4 (12.1) | 129.5 (11.9) | 0.7537 |
| Medication, *n* (%) |  |  |  |  |  |  |  |  |  |
| ARB/ACE inhibitor | 3,633 (38.8) | 1,815 (38.7) | 0.9653 | 1,901 (46.9) | 967 (47.8) | 0.5449 | 1,732 (32.6) | 848 (31.9) | 0.5277 |
| β-blocker | 1,609 (17.2) | 813 (17.4) | 0.7739 | 1,068 (26.4) | 541 (26.7) | 0.7532 | 541 (10.2) | 272 (10.2) | 0.9373 |
| Diuretics | 1,299 (13.9) | 659 (14.1) | 0.7650 | 832 (20.5) | 420 (20.7) | 0.8654 | 467 (8.8) | 239 (9.0) | 0.7954 |
| Statins | 4,616 (49.3) | 2,303 (49.2) | 0.8828 | 2,271 (56.1) | 1,133(56.0) | 0.9206 | 2,345 (44.1) | 1,170 (44.0) | 0.9189 |
| Antithrombotics | 3,871 (41.3) | 1,959 (41.8) | 0.5639 | 2,207 (54.5) | 1,120 (55.3) | 0.5450 | 1,664 (31.3) | 839 (31.6) | 0.7928 |
| Smoking status *n* (%) |  |  | 0.3840 |  |  | 0.7432 |  |  | 0.3911 |
| Non-smoker | 5,440 (58.1) | 2,683 (57.3) |  | 2,560 (63.2) | 1,271 (62.8) |  | 2,880 (54.2) | 1,412 (53.1) |  |
| Ex-smoker | 1,874 (20.0) | 955 (20.4) |  | 739 (18.2) | 374 (18.5) |  | 1,135 (21.3) | 581 (21.9) |  |
| Current-smoker | 2,054 (21.9) | 1,046 (22.3) |  | 751 (18.5) | 380 (18.8) |  | 1,303 (24.5) | 666 (25.0) |  |
| Alcohol, *n* (%) |  |  | 0.969 |  |  | 0.6096 |  |  | 0.6216 |
| No | 5,735 (61.2) | 2,869 (61.3) |  | 2,742 (67.7) | 1,383 (68.3) |  | 2,993 (56.3) | 1,486 (55.9) |  |
| Yes | 3,633 (38.8) | 1,815 (38.8) |  | 1,308 (32.3) | 642 (31.8) |  | 2,325 (43.7) | 1,173 (44.1) |  |
| Physical activity, *n* (%) |  |  | 0.5154 |  |  | 0.4533 |  |  | 0.8344 |
| None | 2,796 (29.8) | 1,404 (30.0) |  | 1,364 (33.7) | 696 (34.4) |  | 1,432 (26.9) | 708 (26.6) |  |
| ≤Twice per week | 1,795 (19.2) | 928 (19.8) |  | 750 (18.5) | 383 (18.9) |  | 1,045 (19.7) | 545 (20.5) |  |
| ≥Three times per week | 4,777 (51.0) | 2,352 (50.2) |  | 1,936 (47.8) | 946 (46.7) |  | 2,841 (53.4) | 1,406 (52.9) |  |

Values are presented as frequencies in numbers (percentages) or means (standard deviation). CVD indicates cardiovascular disease; DPP-4, dipeptidyl peptidase-4; *n*, number; FBS, fasting blood sugar; TZD, thiazolidinedione; BMI, body mass index; SBP, systolic blood pressure; ARB, angiotensin II receptor antagonists; ACE. Angiotensin-converting enzyme.

^a^Cases and controls were matched by the listed variables

^b^*P*-value by generalized estimating equations method

**Table S2.** Baseline characteristics of cases and matched controls, according to the presence or absence of a history of heart failure (HF) by a propensity score matching method (2^nd^ cohort)

| **Characteristics** | **Total** | | | **History of baseline HF** | | | **No history of baseline HF** | | |
| --- | --- | --- | --- | --- | --- | --- | --- | --- | --- |
|  | DPP4i  + MET  (*n* = 9,348) | SU  + MET^a^  (*n* = 4,674) | *P*-value^b^ | DPP4i  + MET  (*n* = 824) | SU  + MET^a^  (*n* = 412) | *P*-value^b^ | DPP4i  + MET  (*n* = 8,524) | SU  + MET^a^  (*n* = 4,262) | *P*-value^b^ |
| Index year, *n* (%) |  |  | 0.9516 |  |  | 0.5065 |  |  | 0.7938 |
| 2008 | 108 (1.2) | 62 (1.3) |  | 8 (1.0) | 4 (1.0) |  | 100 (1.2) | 58 (1.4) |  |
| 2009 | 2,125 (22.7) | 1,065 (22.8) |  | 196 (23.8) | 103 (25.0) |  | 1,929 (22.6) | 962 (22.6) |  |
| 2010 | 2,175 (23.3) | 1,076 (23.0) |  | 211 (25.6) | 110 (26.7) |  | 1,964 (23.0) | 966 (22.7) |  |
| 2011 | 1,906 (20.4) | 928 (19.9) |  | 170 (20.6) | 79 (19.2) |  | 1,736 (20.4) | 849 (19.9) |  |
| 2012 | 1,730 (18.5) | 894 (19.1) |  | 137 (16.6) | 68 (16.5) |  | 1,593 (18.7) | 826 (19.4) |  |
| 2013 | 1,304 (13.9) | 649 (13.9) |  | 102 (12.4) | 48 (11.7) |  | 1,202(14.1) | 601 (14.1) |  |
| Age, mean (SD) | 62.4 (9.1) | 62.5 (9.2) | 0.4312 | 68.0 (9.7) | 68.3 (9.3) | 0.5015 | 61.8 (8.8) | 61.9 (8.9) | 0.1256 |
| Age group, *n* (%) |  |  | 0.3441 |  |  | 0.6004 |  |  | 0.4520 |
| 46–49 | 430 (4.6) | 201 (4.3) |  | 15 (1.8) | 5 (1.2) |  | 415 (4.9) | 196 (4.6) |  |
| 50–59 | 3,607 (38.6) | 1,782 (38.1) |  | 172 (20.9) | 77 (18.7) |  | 3,435 (40.3) | 1,705 (40.0) |  |
| 60–69 | 3,043 (32.6) | 1.538 (32.9) |  | 234 (28.4) | 126 (30.6) |  | 2,809 (33.0) | 1,412 (33.1) |  |
| 70–79 | 1,903 (20.4) | 966 (20.7) |  | 301 (36.5) | 155 (37.6) |  | 1,602 (18.8) | 811 (19.0) |  |
| ≥80 | 365 (3.9) | 187 (4.0) |  | 102 (12.4) | 49 (11.9) |  | 263 (3.1) | 138 (3.2) |  |
| Sex, *n* (%) |  |  | 0.9596 |  |  | 0.9639 |  |  | 0.7389 |
| Male | 5,713 (61.1) | 2,854 (61.1) |  | 357 (43.3) | 181 (43.9) |  | 5,356 (62.8) | 2,673 (62.7) |  |
| Female | 3,635 (38.9) | 1,820 (38.9) |  | 467 (56.7) | 231 (56.1) |  | 3,168 (37.2) | 1,589 (37.3) |  |
| Duration of diabetes, mean (SD), years | 6.4 (3.2) | 6.4 (3.3) | 0.0697 | 7.0 (3.0) | 7.0 (3.2) | 0.8104 | 6.4 (3.2) | 6.3 (3.4) | 0.0697 |
| FBS,  mean(SD),mg/dL | 126.0 (28.9) | 125.9 (31.3) | 0.8432 | 118.0 (25.3) | 117.0 (29.3) | 0.6104 | 126.8 (29.1) | 126.7 (31.4) | 0.8355 |
| Use of TZD, *n* (%) | 400 (4.3) | 195 (4.2) | 0.7636 | 36 (4.4) | 20 (4.9) | 0.7183 | 364 (4.3) | 175 (4.1) | 0.6825 |
| BMI,  mean (SD), kg/m^2^ | 25.3 (3.0) | 25.3 (3.0) | 0.7061 | 25.7 (3.2) | 25.5 (3.1) | 0.4729 | 25.3 (2.9) | 25.3 (3.0) | 0.4609 |
| SBP,  mean (SD), mmHg | 130.5 (12.5) | 130.6 (12.1) | 0.5237 | 132.9 (13.3) | 133.0 (12.6) | 0.7638 | 130.2 (12.3) | 130.4 (12.0) | 0.5262 |
| Medication, *n* (%) |  |  |  |  |  |  |  |  |  |
| ARB/ACE inhibitor | 3,625 (38.8) | 1,797 (38.4) | 0.7075 | 425 (51.6) | 218 (52.9) | 0.6268 | 3,200 (37.5) | 1,579 (37.0) | 0.6047 |
| β-blocker | 1,610 (17.2) | 836 (17.9) | 0.3219 | 289 (35.1) | 151 (36.7) | 0.5927 | 1,321 (15.5) | 685 (16.1) | 0.3879 |
| Diuretics | 1,300 (13.9) | 680 (14.5) | 0.2901 | 314 (38.1) | 163 (39.6) | 0.5170 | 986 (11.6) | 517 (12.1) | 0.3562 |
| Statins | 4,690 (50.2) | 2,289 (49.0) | 0.1772 | 489 (59.3) | 243 (59.0) | 0.9612 | 4,201 (49.3) | 2,046 (48.0) | 0.1720 |
| Antithrombotics | 3,917(41.9) | 1,944 (41.6) | 0.7278 | 514 (62.4) | 263 (63.8) | 0.6246 | 3,403 (39.9) | 1,681 (39.4) | 0.6522 |
| Smoking status *n* (%) |  |  | 0.6222 |  |  | 0.8495 |  |  | 0.5714 |
| Non-smoker | 5,398 (57.7) | 2,689 (57.5) |  | 600 (72.8) | 302 (73.3) |  | 4,798 (56.3) | 2,387 (56.0) |  |
| Ex-smoker | 1,921 (20.5) | 940 (20.1) |  | 128 (15.5) | 63 (15.3) |  | 1,793 (21.0) | 877 (20.6) |  |
| Current-smoker | 2,029 (21.7) | 1,045 (22.4) |  | 96 (11.7) | 47 (11.4) |  | 1,933 (22.7) | 998 (23.4) |  |
| Alcohol, *n* (%) |  |  | 0.6799 |  |  | 0.8553 |  |  | 0.6966 |
| No | 5,753 (61.5) | 2,866 (61.3) |  | 654 (79.4) | 325 (78.9) |  | 5,099 (59.8) | 2,541 (59.6) |  |
| Yes | 3,595 (38.5) | 1,808 (38.7) |  | 170 (20.6) | 87 (21.1) |  | 3,425 (40.2) | 1,721 (40.4) |  |
| Physical activity, *n* (%) |  |  | 0.9803 |  |  | 0.5559 |  |  | 0.8228 |
| None | 2,812 (30.1) | 1,399 (29.9) |  | 351 (42.6) | 169 (41.0) |  | 2,461 (28.9) | 1,230 (28.9) |  |
| ≤Twice per week | 1,813 (19.4) | 920 (19.7) |  | 139 (16.9) | 69 (16.7) |  | 1,674 (19.6) | 851 (20.0) |  |
| ≥Three times per week | 4,723 (50.5) | 2,355 (50.4) |  | 334 (40.5) | 174 (42.2) |  | 4,389 (51.5) | 2,181 (51.2) |  |

Values are presented as frequencies in numbers (percentages) or means (standard deviation). HF indicates heart failure; DPP-4, dipeptidyl peptidase-4; *n* number; FBS, fasting blood sugar; TZD, thiazolidinedione; BMI, body mass index; SBP, systolic blood pressure; ARB, angiotensin II receptor antagonists; ACE. Angiotensin-converting enzyme.

^a^Cases and controls were matched by the listed variables

^b^*P*-value by generalized estimating equations method

**Table S3.** Relative risks of HHF in SU group vs. DPP4i group in the cohort according to history of baseline CKD

| **Study outcomes** | **Total** | | | **History of baseline CKD^a^** | | | | **No history of baseline CKD** | | |
| --- | --- | --- | --- | --- | --- | --- | --- | --- | --- | --- |
|  | SU  + MET  (*n* = 4,718 ) | DPP4i  + MET  (*n* = 9,436 ) | *P*-value | SU  + MET  (*n* = 64 ) | DPP4i  + MET  (*n* = 128 ) | | *P*-value | SU  + MET  (*n* = 4,654 ) | DPP4i  + MET  (*n* = 9,308 ) | *P*-value |
| **HHF** | | | | | | | | | | |
| *N*. of events | 54 | 134 |  | 0 | | 8 |  | 54 | 126 |  |
| Cumulative incidence  at 3 yrs (%)^b^ | 1.29  (0.95-1.76) | 2.29  (1.89-2.77) |  | - | | 8.80  (4.07-18.46) |  | 1.31  (0.96-1.78) | 2.21  (1.81-2.69) |  |
| HR (95% CI)  at 3 yrs ^C^ | 1.00 | 1.85  (1.31-2.60) | 0.0005 | - | | - | - | 1.00 | 1.74  (1.24-2.47) | 0.0016 |
| Cumulative incidence  at 5 yrs (%)^b^ | 2.33  (1.69-3.20) | 5.62  (3.29-9.52) |  | - | | 15.55  (5.89-37.56) |  | 2.35  (1.71-3.24) | 5.49  (3.15-9.46) |  |
| HR (95% CI)  at 5 yrs ^C^ | 1.00 | 1.72  (1.26-2.36) | 0.0007 | - | | - | - | 1.00 | 1.62  (1.18-2.23) | 0.0027 |

All of HHF outcomes were assessed using a Cox proportional hazards models comparing dipeptidyl-peptidase 4 inhibitor with sulfonylurea in combination with metformin after propensity score matching (PMS). PSM was performed by an optimal 2:1 (case:control) matching within a radius of 0.01. CKD indicates chronic kidney disease; DPP-4 inhibitor, dipeptidyl peptidase-4 inhibitor; *N*, number; yrs, years; HR, hazard ratio; CI, confidence interval and HHF, hospitalization for heart failure

**^a^**Baseline CKD was defined by previous diagnosis of ESRD (ICD-10 code N18.0-18.5), hospital visits involving renal dialysis (Z49.1 and Z49.2), kidney transplantation status (Z94.0), procedures for hemodialysis or peritoneal dialysis (O7020, O7030-7034, O7071, and O7072) or surgical procedures for kidney transplantation (R3280).

^b^Cumulative incidence was calculated based on Kaplan-Meier estimation.

^c^*P*-value by cox proportional regression model for clustered data.

**Table S4.** Relative risks of HF and ESRD in SU group vs. DPP4i group in subjects who have never used TZD (1^st^ cohort)

| **Study outcomes** | **Total** | | | **History of baseline CVD** | | | | **No history of baseline CVD** | | |
| --- | --- | --- | --- | --- | --- | --- | --- | --- | --- | --- |
|  | SU  + MET  (*n* = 4,172 ) | DPP4i  + MET  (*n* = 8,344 ) | *P*-value | SU  + MET  (*n* = 1,804 ) | DPP4i  + MET  (*n* = 3,608 ) | | *P*-value | SU  + MET  (*n* = 2,368 ) | DPP4i  + MET  (*n* = 4,736 ) | *P*-value |
| **HHF** | | | | | | | | | | |
| *N*. of events | 58 | 113 |  | 50 | 89 |  | | 8 | 24 |  |
| Cumulative incidence  at 3 yrs (%)^b^ | 1.73  (1.29-2.33) | 2.03  (1.65-2.50) |  | 3.53  (2.58-4.81) | 3.56  (2.82-4.48) |  | | 0.30  (0.12-0.72) | 0.89  (0.56-1.40) |  |
| HR (95% CI)  at 3 yrs ^c^ | 1.00 | 1.33  (0.95-1.86) | 0.0922 | 1.00 | 1.17  (0.82-1.68) | 0.3896 | | 1.00 | 2.86  (1.09-7.50) | 0.0325 |
| Cumulative incidence  at 5 yrs (%)^b^ | 2.86  (2.09-3.90) | 6.09  (3.07-11.89) |  | 5.36  (3.84-7.45) | 11.74  (5.55-23.91) |  | | 0.79  (0.36-1.74) | 1.26  (0.65-2.43) |  |
| HR (95% CI)  at 5 yrs ^c^ | 1.00 | 1.25  (0.92-1.71) | 0.1514 | 1.00 | 1.16  (0.83-1.62) | 0.3952 | | 1.00 | 1.94  (0.88-4.26) | 0.1000 |
| **ESRD events^d^** | | | | | | | | | | |
| *N*. of events/total | 5/3,619 | 13/6,918 |  | 2/1,482 | 9/2,861 |  | | 3/2,137 | 4/4,057 |  |
| Cumulative incidence  at 5 yrs (%)^b^ | 0.31  (0.12-0.82) | 0.42  (0.22-0.80) |  | 0.29  (0.06-1.32) | 0.58  (0.30-1.14) |  | | 0.33  (0.10-1.14) | 0.32  (0.10-1.08) |  |
| HR (95% CI)  at 5 yrs ^c^ | 1.00 | 1.75  (0.63-4.88) | 0.2853 | 1.00 | 3.09  (0.67-14.28) | 0.1482 | | 1.00 | 0.95  (0.21-4.25) | 0.9409 |

**Table S5.** Relative risks of HF and ESRD in SU group vs. DPP4i group in subjects who have never used TZD (2^nd^ cohort)

| **Study outcomes** | **Total** | | | **History of baseline HF** | | | | **No history of baseline HF** | | |
| --- | --- | --- | --- | --- | --- | --- | --- | --- | --- | --- |
|  | SU  + MET  (*n* = 4,197 ) | DPP4i  + MET  (*n* = 8,394 ) | *P*-value | SU  + MET  (*n* = 375 ) | DPP4i  + MET  (*n* = 750 ) | | *P*-value | SU  + MET  (*n* = 3,822 ) | DPP4i  + MET  (*n* = 7,644 ) | *P*-value |
| **HHF** | | | | | | | | | | |
| *N*. of events | 56 | 112 |  | 35 | | 65 |  | 21 | 47 |  |
| Cumulative incidence  at 3 yrs (%)^a^ | 1.62  (1.19-2.19) | 2.03  (1.65-2.50) |  | 10.90  (7.64-15.42) | | 12.72  (9.85-16.35) |  | 0.67  (0.39-1.13) | 0.96  (0.69-1.33) |  |
| HR (95% CI)  at 3 yrs ^b^ | 1.00 | 1.40  (0.999-1.95) | 0.0510 | 1.00 | | 1.27  (0.83-1.95) | 0.2755 | 1.00 | 1.67  (0.95-2.92) | 0.0755 |
| Cumulative incidence  at 5 yrs (%)^a^ | 2.78  (2.01-3.83) | 5.88  (3.10-11.01) |  | 17.93  (11.90-26.52) | | 31.60  (13.23-63.80) |  | 1.31  (0.77-2.21) | 3.15  (1.16-8.39) |  |
| HR (95% CI)  at 5 yrs ^b^ | 1.00 | 1.32  (0.97-1.81) | 0.0809 | 1.00 | | 1.20  (0.80-1.81) | 0.7789 | 1.00 | 1.51  (0.91-2.51) | 0.1087 |
| **ESRD events**^c^ | | | | | | | | | | |
| *N*. of events | 5/3,669 | 15/6,966 |  | 0/306 | | 4/568 |  | 5/3,363 | 11/6,398 |  |
| Cumulative incidence  at 5 yrs (%)^a^ | 0.24  (0.10-0.61) | 0.59  (0.30-1.17) |  | - | | 3.03  (0.85-10.46) |  | 0.27  (0.10-0.67) | 0.37  (0.18-0.76) |  |
| HR (95% CI)  at 5 yrs ^b^ | 1.00 | 1.96  (0.71-5.40) | 0.1937 | - | | - | - | 1.00 | 1.42  (0.49-4.10) | 0.5148 |

**Table S6.** Relative risks of HHF in SU group vs. each DPP4i group in both cohort.

| 1^ST^ cohort | Total | | History of baseline CVD | | No history of CVD | |
| --- | --- | --- | --- | --- | --- | --- |
| **HHF** | SU  + MET | DPP4i  + MET | SU  + MET | DPP4i  + MET | SU  + MET | DPP4i  + MET |
| Vildagliptin |  |  |  |  |  |  |
| N. of event/Total | 32/2,092 | 34/2,400 | 29/923 | 29/1,071 | 3/1,169 | 5/1,329 |
| HR (95% CI) at 3 years | 1.00 | 1.29 (0.77-2.16) | 1.00 | 1.16 (0.67-2.00) | 1.00 | 5.15 (0.60-44.18) |
| HR (95% CI) at 5 years | 1.00 | 1.14 (0.70-1.85) | 1.00 | 1.10 (0.65-1.85) | 1.00 | 1.77 (0.43-7.24) |
| Sitagliptin |  |  |  |  |  |  |
| N. of event/Total | 57/4,033 | 87/5,868 | 49/1,754 | 70/2,577 | 8/2,279 | 17/3,291 |
| HR (95% CI) at 3 years | 1.00 | **1.48 (1.03-2.11)** | 1.00 | 1.31 (0.88-1.92) | 1.00 | **2.99 (1.10-8.11)** |
| HR (95% CI) at 5 years | 1.00 | 1.35 (0.98-1.88) | 1.00 | 1.27 (0.88-1.82) | 1.00 | 1.94 (0.88-4.28) |
| Saxagliptin |  |  |  |  |  |  |
| N. of event/Total | 3/177 | 1/179 | 2/73 | 0/73 | 1/104 | 1/106 |
| HR (95% CI) at 3 years | 1.00 | 0.69 (0.08-6.06) | - | - | 1.00 | 2.17 (0.23-20.59) |
| Linagliptin |  |  |  |  |  |  |
| N. of event/Total | 8/785 | 5/834 | 6/288 | 3/300 | 2/497 | 2/534 |
| HR (95% CI) at 5 years | 1.00 | **3.97 (1.06-14.89)** | 1.00 | 2.68 (0.63-11.32) | - | - |
| Zemigliptine |  |  |  |  |  |  |
| N. of event/Total | 1/87 | 1/87 | 0/29 | 1/29 | 1/58 | 0/58 |
| 2^nd^ cohort | Total | | History of baseline HF | | No history of HF | |
| **HHF** | SU  + MET | DPP4i  + MET | SU  + MET | DPP4i  + MET | SU  + MET | DPP4i  + MET |
| Vildagliptin |  |  |  |  |  |  |
| N. of event/Total | 30/2,103 | 37/2,406 | 15/190 | 21/228 | 15/1,913 | 16/2,178 |
| HR (95% CI) at 3 years | 1.00 | 1.33 (0.81-2.20) | 1.00 | 1.57 (0.79-3.14) | 1.00 | 1.17 (0.56-2.47) |
| HR (95% CI) at 5 years | 1.00 | 1.29 (0.80-2.07) | 1.00 | 1.51 (0.78-2.91) | 1.00 | 1.10 (0.54-2.23) |
| Sitagliptin |  |  |  |  |  |  |
| N. of event/Total | 58/4,040 | 84/5,856 | 39/359 | 49/524 | 19/3,681 | 35/5,332 |
| HR (95% CI) at 3 years | 1.00 | **1.47 (1.02-2.10)** | 1.00 | 1.32 (0.83-2.09) | 1.00 | **1.88 (1.001-3.54)** |
| HR (95% CI) at 5 years | 1.00 | 1.28 (0.92-1.78) | 1.00 | 1.12 (0.73-1.72) | 1.00 | 1.66 (0.95-2.90) |
| Saxagliptin |  |  |  |  |  |  |
| N. of event/Total | 2/170 | 3/173 | 1/11 | 0/11 | 1/159 | 3/162 |
| Linagliptin |  |  |  |  |  |  |
| N. of event/Total | 9/783 | 5/824 | 4/51 | 2/54 | 5/732 | 3/770 |
| HR (95% CI) at 5 years | 1.00 | 1.36 (0.44-4.18) | 1.00 | 1.34 (0.19-9.71) | 1.00 | 1.33 (0.34-5.22) |
| Zemigliptine |  |  |  |  |  |  |
| N. of event/Total | 4/89 | 2/89 | 3/7 | 1/7 | 1/82 | 1/82 |
| HR (95% CI) at 5 years | 1.00 | 1.57 (0.23-10.85) | 1.00 | 1.29 (0.05-32.80) | 1.00 | 2.15 (0.27-16.97) |

**Table S7.** Relative risks of total cardiovascular disease, heart failure, non-fatal myocardial infarction, and ischemic stroke in patients treated with sulfonylurea plus metformin vs. DPP-4 inhibitor plus metformin by using inverse probability of treatment weighting (IPTW) (1st cohort)

| **Study outcomes** | **Total** | | | **History of baseline CVD** | | | | | **No history of baseline CVD** | | | |
| --- | --- | --- | --- | --- | --- | --- | --- | --- | --- | --- | --- | --- |
|  | SU  + MET  (n=23,411) | DPP4i  + MET (n=23,600) | *P*-value | SU  + MET  (n=10,589) | DPP4i  + MET (n=10,823) | | | *P*-value | SU  + MET  (n=12,822) | DPP4i  + MET (n=12,777) | | *P*-value |
| **Composite CVD events^a^** | | | | | | | | | | | | |
| *N*. of events | 1500 | 1150 |  | 1223 | | 902 | |  | 277 | 248 | |  |
| Cumulative incidence at 3 yrs (%)^b^ | 8.58 (7.55–9.73) | 7.92 (7.31–8.58) |  | 15.06 (12.94–17.50) | | 13.22 (12.15–14.38) | |  | 2.74 (2.07–3.61) | 3.47 (2.89–4.16) | |  |
| HR (95% CI) at 3 yrs ^c^ | 1.00 | 0.99 (0.85–1.16) | 0.9317 | 1.00 | | 0.97 (0.81–1.16) | | 0.7107 | 1.00 | 1.20 (0.88–1.64) | | 0.2477 |
| Cumulative incidence at 5 yrs (%)^b^ | 11.88 (10.11–13.93) | 14.71 (11.99–17.99) |  | 19.01 (16.25–22.19) | | 25.10 (19.97–31.28) | |  | 5.32 (3.49–8.07) | 5.07 (4.04–6.37) | |  |
| HR (95% CI) at 5 yrs ^c^ | 1.00 | 1.01 (0.87, 1.17) | 0.8944 | 1.00 | | 1.00 (0.84–1.18) | | 0.9661 | 1.00 | 1.16 (0.87–1.55) | | 0.3108 |
| **IHD** | | | | | | | | | | | | |
| *N*. of events | 701 | 534 |  | 581 | | 409 | |  | 120 | 125 | |  |
| Cumulative incidence at 3 yrs (%)^b^ | 4.27 (3.53–5.15) | 3.73 (3.31–4.21) |  | 7.73 (6.14–9.70) | | 6.30 (5.54–7.18) | |  | 1.25 (0.83–1.89) | 1.63 (1.25–2.13) | |  |
| HR (95% CI) at 3 yrs ^c^ | 1.00 | 0.99 (0.79–1.23) | 0.9009 | 1.00 | | 0.94 (0.73–1.21) | | 0.6414 | 1.00 | 1.29 (0.82–2.03) | | 0.2665 |
| Cumulative incidence at 5 yrs (%)^b^ | 5.69 (4.54–7.13) | 5.54 (4.78–6.41) |  | 9.67 (7.64–12.19) | | 8.72 (7.51–10.11) | |  | 2.17 (1.16–4.04) | 2.91 (2.06–4.11) | |  |
| HR (95% CI) at 5 yrs ^c^ | 1.00 | 1.01 (0.82–1.25) | 0.9221 | 1.00 | | 0.96 (0.76–1.22) | | 0.7503 | 1.00 | 1.34 (0.88–2.05) | | 0.1740 |
| **IS** | | | | | | | | | | | | |
| *N*. of events | 469 | 344 |  | 360 | | 275 | |  | 109 | 69 | |  |
| Cumulative incidence  at 3 yrs (%)^b^ | 2.66 (2.10–3.36) | 2.43 (2.09–2.83) |  | 4.45 (3.32–5.97) | | 4.15 (3.53–4.88) | |  | 1.07 (0.67–1.68) | 0.99 (0.69–1.41) | |  |
| HR (95% CI)  at 3 yrs ^c^ | 1.00 | 0.94 (0.72–1.22) | 0.6256 | 1.00 | | 0.97 (0.71–1.32) | | 0.8502 | 1.00 | 0.86 (0.51–1.46) | | 0.5775 |
| Cumulative incidence  at 5 yrs (%)^b^ | 3.91 (2.80–5.47) | 5.80 (3.81–8.77) |  | 5.52 (4.09–7.44) | | 10.62 (6.67–16.69) | |  | 2.52 (1.24–5.08) | | 1.36 (0.91–2.01) |  |
| HR (95% CI)  at 5 yrs ^c^ | 1.00 | 1.04 (0.77–1.40) | 0.8104 | 1.00 | | 1.04 (0.77–1.40) | | 0.8104 | 1.00 | 1.04 (0.78–1.40) | | 0.7842 |
| **HHF** | | | | | | | | | | | | |
| *N*. of events | 296 | 323 |  | 260 | | 263 |  | | 37 | 60 | |  |
| Cumulative incidence  at 3 yrs (%)^b^ | 1.53 (1.12–2.10) | 2.23 (1.91–2.60) |  | 2.93 (2.03–4.24) | | 3.81 (3.24–4.48) |  | | 0.28 (0.12–0.65) | 0.91 (0.63–1.31) | |  |
| HR (95% CI)  at 3 yrs ^c^ | 1.00 | 1.59 (1.16–2.17) | 0.0041 | 1.00 | 1.44 (1.03–2.03) | | 0.0348 | | 1.00 | 3.07 (1.50–6.32) | | 0.0023 |
| Cumulative incidence  at 5 yrs (%)^b^ | 2.70 (1.93–3.76) | 4.42 (2.80–6.95) |  | 4.79 (3.22–7.09) | 8.09 (4.84–13.36) | |  | | 0.73 (0.34–1.57) | 1.10 (0.69–1.75) | |  |
| HR (95% CI)  at 5 yrs ^c^ | 1.00 | 1.36 (1.00–1.87) | 0.0518 | 1.00 | 1.36 (1.00–1.87) | | 0.0518 | | 1.00 | 1.35 (0.99–1.85) | | 0.0614 |
| **CVD death** | | | | | | | | | | | | |
| *N*. of events | 202 | 88 |  | 169 | 69 | |  | | 33 | 19 | |  |
| Cumulative incidence  at 3 yrs (%)^b^ | 1.17 (0.81–1.67) | 0.60 (0.45–0.82) |  | 2.17 (1.41–3.33) | 1.04 (0.75–1.43) | |  | | 0.26 (0.11–0.61) | 0.24 (0.12–0.48) | |  |
| HR (95% CI)  at 3 yrs ^c^ | 1.00 | 0.56 (0.30–1.04) | 0.00671 | 1.00 | 0.53 (0.26–1.06) | | 0.0729 | | 1.00 | 0.82 (0.26–2.65) | | 0.7407 |
| Cumulative incidence  at 5 yrs (%)^b^ | 1.61 (1.12–2.31) | 1.18 (0.80–1.74) |  | 2.79 (1.81–4.28) | 2.13 (1.39–3.27) | |  | | 0.52 (0.23–1.19) | 0.33 (0.17–0.64) | |  |
| HR (95% CI)  at 5 yrs ^c^ | 1.00 | 0.59 (0.31–1.12) | 0.1046 | 1.00 | 0.59 (0.31–1.12) | | 0.1046 | | 1.00 | 0.59 (0.31–1.11) | | 0.0988 |

All cardiovascular outcomes were assessed using a Cox proportional hazards models comparing dipeptidyl-peptidase 4 inhibitor with sulfonylurea in combination with metformin after propensity score matching. CVD indicates cardiovascular disease; DPP-4 inhibitor, dipeptidyl peptidase-4 inhibitor; *N*, number; yrs, years; HR, hazard ratio; CI, confidence interval; IHD, ischemic heart disease, IS, ischemic stroke, HF, heart failure

^a^Any occurrence of IHD, IS, HF, or CVD death.

^b^Cumulative incidence was calculated based on Kaplan-Meier estimation.

^c^*P*-value by weighted cox proportional regression model.

**Table S8.** Proportion of DPP-4 inhibitors prescribed in the DPP-4 inhibitor group

|  | Patients  (*n* = 16,803) | Proportion (%) |
| --- | --- | --- |
| Sitagliptin | 10,162 | 60.5% |
| Vildagliptin | 4,332 | 25.78% |
| Linagliptin | 1,792 | 10.66% |
| Saxagliptin | 331 | 1.97% |
| Gemigliptin | 186 | 1.11% |

**Table S9.** List of codes for diagnoses, procedures and medications used in this study

| Diagnosis or procedure | Corresponding codes |
| --- | --- |
| Cardiovascular events |  |
| Cardiovascular disease | ICD-10 codes I00-I99 |
| Ischemic heart disease | ICD-10 codes I20-I25 |
| Ischemic stroke | ICD-10 codes I63-I66 |
| Heart failure | ICD-10 codes I50, I42, I43 |
| Coronary revascularization | procedure code HA607, O1641, O1642, O1647, OA641, OA642, OA647, M6551, M6552, M6561, M6562, M6563, M6564, M6571, M6572 |
| Renal events |  |
| End stage renal disease | ICD-10 codes N18.0 and N18.5 |
| Hospital visits involving renal dialysis | Z49.1-Z49.2 |
| Kidney transplantation status | Z94.0 |
| Procedure for hemodialysis or peritoneal dialysis | O7020, O7030-7034, O7071, and O7072 |
| Procedure for kidney transplantations | R3280 |
| Medication properties (including combination) |  |
| Metformin | metformin hydrochloride |
| Sulfonylurea | Glibenclamide, gliclazide, glimepiride, glipizide |
| Thiazolidinedione | pioglitazone hydrochloride, lobeglitazone sulfate |
| DPP-4 inhibitor | vildagliptin, sitagliptin, saxagliptin, linagliptin, gemigliptin tartrate, alogliptin, teneligliptin hydrobromide, anagliptin, evogliptin |
| Calcium channel blocker | amlodipine, S-amlodipine, barnidipine, benidipine, cilnidipine,  felodipine, isradipine, lacidipine, lercanidipine, manidipine,  nicardipine, nifedipine, nimodipine, nisoldipine,  efonidipine, lercanidipine, verapamil, diltiazem, flunarizine |
| Beta blocker | amosulalol, atenolol, arotinolol, betaxolol, bevantolol, bisoprolol, carteolol, carvedilol, celiprolol, esmolol, labetalo, metoprolol,  nadolol, nebivolol, propranolol, S-atenolol, sotalol |
| ACE inhibitor | alacepril, benazepril, captopril, cilazapril, enalapril, fosinopril, imidapril, lisinopril, moexipril, perindopril, quinapril, ramipril, temocapril, zofenopril |
| ARB | candesartan, irbesartan, losartan, valsartan, telmisartan, eprosartan,  olmesartan, fimasartan, ambrisentan |
| Alpha blokcer | doxazosin, phenoxybenzamine |
| Diuretics | amiloride, chlorthalidone, furosemide, hydrochlorothiazide, indapamide, metolazone, torsemide, xipamide |
| Aldacton antagonists | spironolactone |
| Statins | atorvastatin, fluvastatin, lovastatin, pravastatin sodium, pitavastatin, rosuvastatin, simvastatin |
| Antithrombotic agents | anagrelide, aspirin, apixaban, cilostazol, clopidogrel, dabigatran, edoxaban, ozagrel, prasugrel, rivaroxaban, sarpogrelate, ticagrelor, ticlopidine, warfarin |
|  |  |

ICD-10, International Classification of Diseases, 10th revision; ACE inhibitor, angiotensin-converting-enzyme inhibitor; ARB, angiotensin II receptor antagonists
