# Supplementary material for: Dipeptidyl peptidase-4 inhibitor compared with sulfonylurea in combination with metformin: cardiovascular and renal outcomes in a propensity-matched cohort study
Source: Cardiovasc Diabetol. 2019 Mar 11;18:28. doi: 10.1186/s12933-019-0835-z (PMC6410523; doi:10.1186/s12933-019-0835-z)
Supplement: Supplementary file 2 — Additional file 2: Figure S1. Disposition of study subjects. Figure S2. Comparison of cumulative incidence for CVD outcomes according to the baseline HF. (A) Incidence of hospitalization for heart failure. (B) Incidence for end-stage renal disease events. [file 12933_2019_835_MOESM2_ESM.pdf]

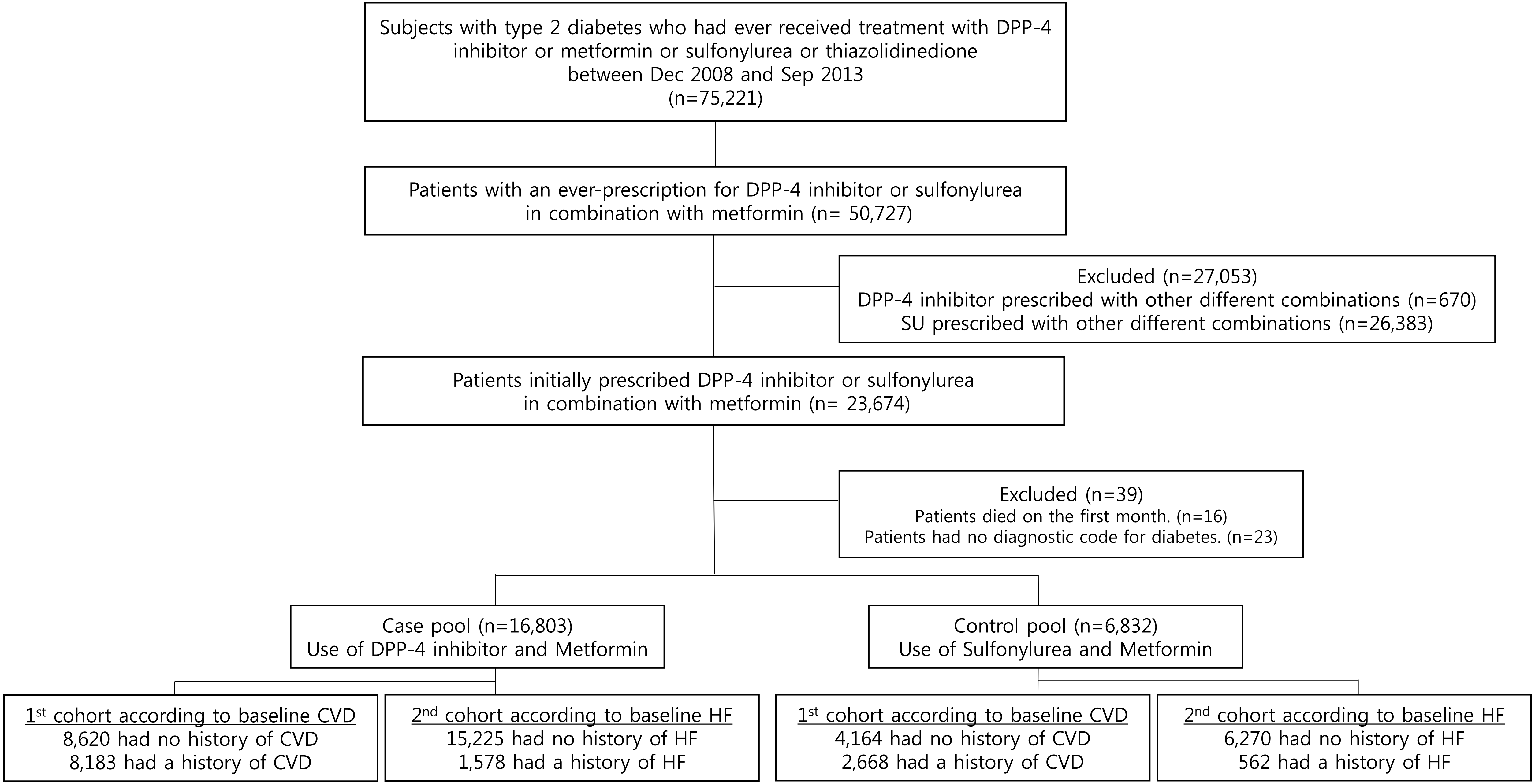

Figure S1. Disposition of study subjects

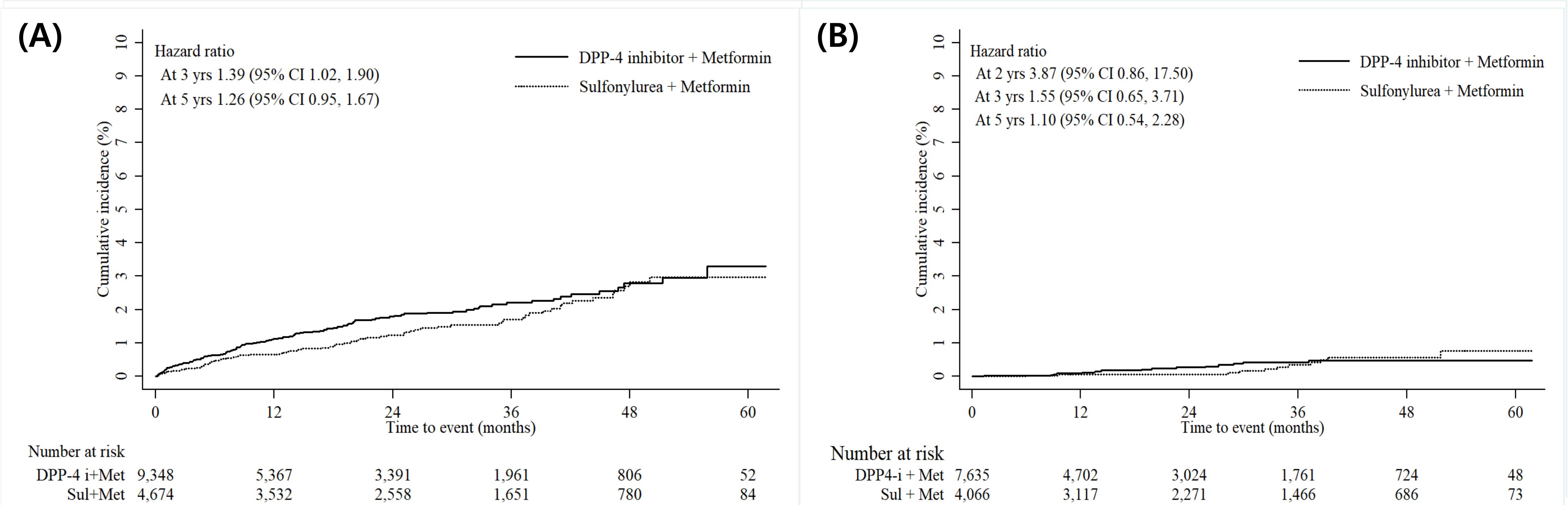

Figure S2. Comparison of cumulative incidence for CVD outcomes according to the baseline HF. (A) Incidence of hospitalization for heart failure. (B) Incidence for end-stage renal disease events.
